# Supplementary material for: Association between oral microbiome and seven types of cancers in East Asian population: a two-sample Mendelian randomization analysis
Source: Front Mol Biosci. 2023 Nov 21;10:1327893. doi: 10.3389/fmolb.2023.1327893 (PMC10702768; doi:10.3389/fmolb.2023.1327893)
Supplement: Supplementary file 1 [file DataSheet1.ZIP › Supplementary Materials/MR plots of saliva/saliva═╝/Pancreatic cancer/pheno.333_to_pancreatic cancer_leave_one_out.pdf]

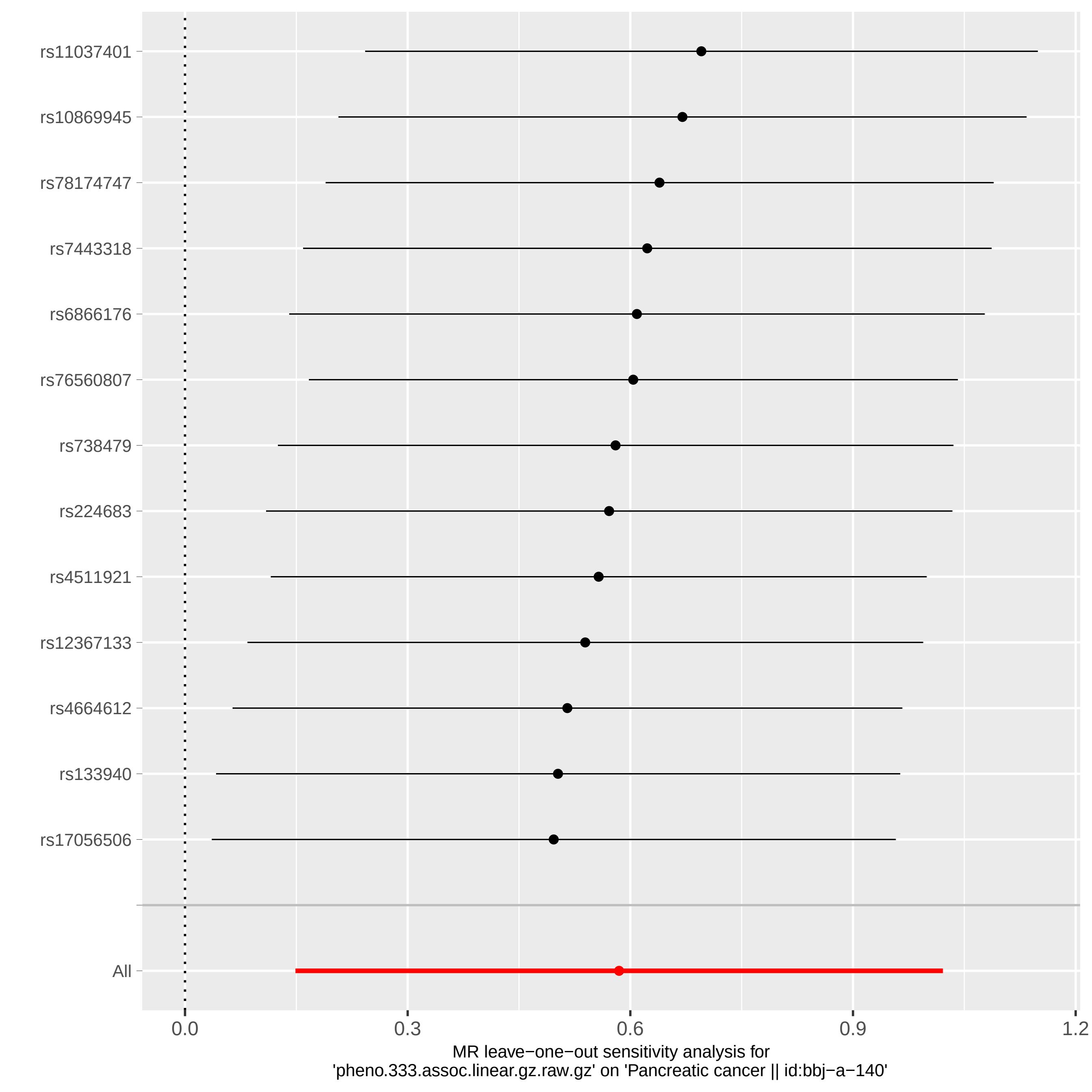

rs11037401
rs10869945
rs78174747
rs7443318
rs6866176
rs76560807
rs738479
rs224683
rs4511921
rs12367133
rs4664612
rs133940
rs17056506
All
0.0
0.3
0.6
0.9
1.2
MR leave−one−out sensitivity analysis for
'pheno.333.assoc.linear.gz.raw.gz' on 'Pancreatic cancer || id:bbj−a−140'
